# Betaine and Trimethylamine-*N*-Oxide as Predictors of Cardiovascular Outcomes Show Different Patterns in Diabetes Mellitus: An Observational Study

**DOI:** 10.1371/journal.pone.0114969

**Published:** 2014-12-10

**Authors:** Michael Lever, Peter M. George, Sandy Slow, David Bellamy, Joanna M. Young, Markus Ho, Christopher J. McEntyre, Jane L. Elmslie, Wendy Atkinson, Sarah L. Molyneux, Richard W. Troughton, Christopher M. Frampton, A. Mark Richards, Stephen T. Chambers

**Affiliations:** 1 Clinical Biochemistry Unit, Canterbury Health Laboratories, Christchurch, New Zealand; 2 Department of Pathology, University of Otago Christchurch, Christchurch, New Zealand; 3 The Christchurch Heart Institute, Department of Medicine, University of Otago Christchurch, Christchurch, New Zealand; University of Messina, Italy

## Abstract

**Background:**

Betaine is a major osmolyte, also important in methyl group metabolism. Concentrations of betaine, its metabolite dimethylglycine and analog trimethylamine-*N*-oxide (TMAO) in blood are cardiovascular risk markers. Diabetes disturbs betaine: does diabetes alter associations between betaine-related measures and cardiovascular risk?

**Methods:**

Plasma samples were collected from 475 subjects four months after discharge following an acute coronary admission. Death (n = 81), secondary acute MI (n = 87), admission for heart failure (n = 85), unstable angina (n = 72) and all cardiovascular events (n = 283) were recorded (median follow-up: 1804 days).

**Results:**

High and low metabolite concentrations were defined as top or bottom quintile of the total cohort. In subjects with diabetes (n = 79), high plasma betaine was associated with increased frequencies of events; significantly for heart failure, hazard ratio 3.1 (1.2–8.2) and all cardiovascular events, HR 2.8 (1.4–5.5). In subjects without diabetes (n = 396), low plasma betaine was associated with events; significantly for secondary myocardial infarction, HR 2.1 (1.2–3.6), unstable angina, HR 2.3 (1.3–4.0), and all cardiovascular events, HR 1.4 (1.0–1.9). In diabetes, high TMAO was a marker of all outcomes, HR 2.7 (1.1–7.1) for death, 4.0 (1.6–9.8) for myocardial infarction, 4.6 (2.0–10.7) for heart failure, 9.1 (2.8–29.7) for unstable angina and 2.0 (1.1–3.6) for all cardiovascular events. In subjects without diabetes TMAO was only significant for death, HR 2.7 (1.6–4.8) and heart failure, HR 1.9 (1.1–3.4). Adding the estimated glomerular filtration rate to Cox regression models tended to increase the apparent risks associated with low betaine.

**Conclusions:**

Elevated plasma betaine concentration is a marker of cardiovascular risk in diabetes; conversely low plasma betaine concentrations indicate increased risk in the absence of diabetes. We speculate that the difference reflects control of osmolyte retention in tissues. Elevated plasma TMAO is a strong risk marker in diabetes.

## Introduction

Betaine (also known as *N,N,N*-trimethylglycine, TMG or glycine betaine) is an essential metabolite, either synthesized from choline in the body or derived directly from the diet [1], [2]. It has two main roles in the human body: firstly, it is an osmolyte accumulated to multi-millimolar concentrations in many tissues, especially the kidneys and liver, where it controls cell volume, stabilizes proteins and protects against premature apoptosis. Secondly, betaine is an important source of methyl groups. The reaction catalyzed by betaine-homocysteine methyltransferase (BHMT) remethylates homocysteine to methionine and therefore is an alternative to methionine synthase [1]–[3]. Betaine metabolism is disturbed in diabetes with plasma concentrations and urinary excretions of betaine and its metabolites more variable than in the healthy population [4], and about a third of patients with diabetes excrete abnormal (above the 97.5 percentile for a healthy adult population) amounts of betaine [5]–[7]. The expression of the key enzyme, BHMT, is modulated by diabetes probably through insulin repression [8], and this may explain some of the variability in plasma betaine and betaine-related metabolites, but not the urine results, so the disturbances in betaine metabolism in diabetes is probably multifactorial.

There is evidence of a complex relationship between plasma betaine levels and cardiovascular health. Both low and high levels of plasma betaine could be markers of an underlying tissue betaine insufficiency [9]–[11]; this association appears to be stronger in diabetes [11]. Low plasma levels may reflect a dietary deficiency of betaine and/or choline, excessive loss of betaine, or defective conversion of choline to betaine [2]. High plasma levels, on the other hand, may be the result of defective control of the efflux of betaine from cells, resulting in a similar tissue insufficiency [11]. Thus it is worth considering the possibility that tissue betaine insufficiency is a cardiovascular risk factor.

Betaine metabolism depends on the BHMT-catalyzed reaction with homocysteine, and elevated plasma homocysteine has been strongly linked to an increased risk for cardiovascular events in a number of studies [12]–[14]. Although supplementation with folate, vitamin B_12_ and vitamin B_6_ is effective in lowering plasma homocysteine levels, these supplements have not been observed to have any effect on the risk of secondary coronary events [15]–[17]. Since betaine insufficiency has also been associated with raised homocysteine levels in a number of populations, especially those with altered folate status [2], [3], it is possible that an underlying betaine insufficiency is a reason why no mean overall benefit has been observed in randomized trials of vitamin supplementation in patients with elevated homocysteine.

Both plasma betaine concentrations and urinary betaine excretion are under homeostatic control [18]–[20], however, betaine excretion tends to be abnormally low or abnormally high in subjects with underlying disease processes, such as renal failure, and especially in diabetes [2], [7], [19]. The immediate product of betaine metabolism, *N,N*-dimethylglycine (DMG), has a particularly strong relationship with cardiovascular events [11], [21], [22]; the only known source of DMG is from betaine metabolism.

Recently, there have been several reports that plasma concentrations of the betaine analog, trimethylamine *N*-oxide (TMAO), are predictive of cardiovascular events and that TMAO may be directly atherogenic [23], [24]. TMAO has previously been associated with renal impairment [25], [26], a common complication of diabetes. The aim of this study was to investigate the role of these different metabolites in predicting cardiovascular events in a high-risk population over a longer period of time than previously reported [11], including TMAO in the metabolites studied. Our earlier analysis [11] suggested that diabetes could affect the risks associated with plasma betaine concentrations, and here we evaluate the effect of diabetes on the predictive power of both betaine and the betaine-related metabolites.

## Methods

### Ethics

All subjects gave written informed consent. The Canterbury Ethics Committee approved the parent study (CTY02/02/018), and approval for the betaine and related markers sub-study was an amendment approved by its successor, the Upper South B Regional Ethics Committee.

### Subjects

Subjects (**Table 1**) were recruited from the Coronary Disease Cohort Study (CDCS), trials registry number ACTRN12605000431628, that was conducted by the Christchurch Heart Institute Group, University of Otago, Christchurch [27]. Inclusion eligibility was based on hospitalization following an acute coronary event, defined as ischemic chest pain plus one or more of a) ECG changes (ST segment depression or elevation of at least 0.5 mm, T-wave inversion of at least 3 mm in at least 3 leads, or left bundle branch block), b) elevated levels of cardiac markers, c) a history of coronary disease, or d) age of at least 65 years in patients with diabetes or vascular disease [28]. Exclusion criteria were severe co-morbidity limiting life expectancy to less than 3 years and inability to provide written consent. Eligible patients (502) were recruited from the CDCS cohort for this sub-study, and 475 plasma samples were collected at the four-month post-discharge outpatient clinic visit. Of these, 79 (16.6%) had clinically-diagnosed diabetes (Table 1), all with Type 2 diabetes. Diabetes was defined by a prior clinical diagnosis of diabetes, prescriptions of antidiabetic medications or fasting plasma glucose levels >7.0 mmol/L.

**Table 1:** Characteristics of the study cohort.

	Without Diabetes	With diabetes
Number	396	79
Median age (total range)	68 (55−93)	74 (47−87)
Gender (M/F)	288/108	58/21
BMI (median, IQ range)	26.2 (23.9−29.4)	28.1 (25.4−31.2)
eGFR (median, IQ range)	65.5 (52.0−75.0)	62.0 (50.0−72.8)
Follow-up time/time to death (median, IQ range) days	1811 (1511−2025)	1758 (1331−1935)
Acute MI, n (%)	65 (16%)	22 (28%)
Admission for heart failure, n (%)	62 (16%)	23 (29%)
Deaths (all-causes), n (%)	62 (16%)	19 (24%)
Unstable angina, n (%)	56 (14%)	16 (20%)
All cardiovascular disease, n (%)	227 (57%)	56 (71%)
Plasma homocysteine (µmol/L, median, IQ range)	12.5 (10.5−15.8)	13.7 (10.5−17.4)
Plasma betaine (µmol/L, median, IQ range)	46.8 (35.7−58.4)	43.2 (36.2−53.4)
Plasma dimethylglycine (µmol/L, median, IQ range)	3.7 (2.6−5.2)	4.2 (2.5−5.9)
Plasma TMAO (µmol/L, median, IQ range)	4.8 (3.0−9.1)	7.5 (4.4−12.1)*

All Type 2 diabetes. TMAO: Trimethylamine *N*-oxide. Betaine data adjusted for gender difference. IQ range: interquartile range.

*Significantly different (p<0.001, Mann-Whitney Rank Sum Test); TMAO data on 74 subjects with diabetes and 381 subjects without diabetes. Other differences not statistically significant (p>0.05). BMI: body mass index. eGFR: estimated glomerular filtration rate. MI: myocardial infarction.

### Follow up

Subjects in this sub-study were followed up for a median time of 1804 days (IQ range: 1467–2015), similar in both subjects with and without diabetes (Table 1). Follow up secondary events captured included all hospitalizations, and recorded all relevant World Health Organization International Classification of Diseases (ICD) discharge codes. Mortality data was taken from the New Zealand national death register.

### Analytical methods

Betaine, *N,N*-dimethylglycine, homocysteine and standard biochemical measures were performed on the samples within a few days of sample collection, with plasma specimens stored at −20C before analysis. TMAO was measured on stored plasma samples after 5 to 8 years storage. Most samples were stored at −85°C, but some aliquots were stored at −20°C; a comparison was made using 16 specimens where two aliquots were stored at different temperatures to confirm that the storage conditions did not affect the results. Specimens were not located from 5 subjects (2 male, 3 female) with diabetes and 15 subjects without diabetes (14 male, 1 female), so that the TMAO analyses were carried out on a slightly smaller sub-cohort than for betaine. The characteristics of this smaller cohort were statistically indistinguishable from those reported in Table 1.

Betaine and DMG were measured in plasma by extraction with a 9∶1 v/v mixture of acetonitrile and methanol (50 µL plasma to 1 mL solvent mixture), followed by the HPLC separation of their 2-naphthacyl derivatives on Merck Aluspher alumina columns [29], [30]. Data on normal subjects have been reviewed elsewhere [2]. Plasma homocysteine was measured by fluorescence polarization on an Abbott IMX Analyzer (Abbott Laboratories USA). Other biochemical measures in plasma were carried out using an Abbott ARCHITECT ci8200 Analyzer (Abbott Laboratories) by standard procedures in an International Accreditation New Zealand accredited laboratory. Glomerular filtration rate (eGFR) data was supplied by the parent study organizers, who had estimated it by the IDMS-traceable MDRD Study equation [31].

Trimethylamine *N*-oxide (TMAO) was obtained from Sigma (St Louis, MO, USA) as its dihydrate, and D_9_-TMAO was obtained from Cambridge Isotopes (Andover, MA, USA) for use as an isotopically labelled internal standard. Samples (and calibration standards) (50 µL) were extracted into an extraction solvent (1 mL) made by mixing 9 volumes of acetonitrile with 1 volume of methanol; to this mixture was added deuterated internal standard, D_9_-TMAO, (10 µmol/L). Samples were then vortex mixed and centrifuged for 5 min at 13000×g. TMAO was quantified using an Agilent 6120 single quadrupole mass spectrometer with an electrospray ion source connected to an Agilent 1200 Series HPLC system. TMAO was measured in positive ion mode monitoring at m/z 76.1, and m/z 85.2 for D_9_-TMAO. A 100×2.1 mm, 4 µm Cogent Diamond Hydride (Microsolv Technologies, NJ, USA) silica column was used for the chromatographic separation. Mobile phase A contained 50% water and 50% acetonitrile containing 10 mmol/L ammonium formate and 10 mmol/L formic acid. Mobile phase B contained 90% acetonitrile and 10% water. The gradient went from 50% A to 100% A over 7 minutes. The flow rate was 0.3 mL/min, the injection volume was 10 µL and the oven temperature was 40°C. The capillary voltage was set to 3500 V, the nebulizer pressure was 35 psig, the drying gas flow rate was 9.0 L/min, and the drying gas temperature was 275°C.

### Statistics

Statistical analyses were carried out using SPSS 20.0 and SigmaPlot 11.2, with p<0.05 accepted as indicating statistical significance. Because previous evidence has suggested both high and low levels of plasma betaine are indicative of a betaine insufficiency [11], [18], the subjects were split into quintiles for all the variables being investigated. The top and bottom 20% of results were independently compared to the middle 60% for the five secondary outcomes: death (all cause), acute MI, heart failure, unstable angina and all cardiovascular events. “All cardiovascular events” included hospitalisation for acute MI, heart failure, unstable angina, cerebrovascular accident, transient ischemic attack, and other cerebrovascular disease. Kaplan-Meier plots were generated for each event and the quintile groups were compared by using log-rank tests. Plasma betaine and plasma DMG were gender-adjusted over the whole cohort, consistent with the methods described in previous studies [2], [22]. The mean male betaine concentration was 21.5% higher, and the mean male DMG concentration was 9.7% higher, than in females. Female values were therefore multiplied by the ratio of the means and the adjusted female values combined with the male values in further statistical analyses.

Subjects with type 2 diabetes (n = 79) and those without diabetes (n = 396) were analyzed separately, but the same cut-offs for high (top quintile) and low (bottom quintile) plasma concentrations were used consistently, based on quintiles of the total population.

To calculate hazard ratios, a “high plasma betaine” group was defined as the top quintile (in the total cohort) of gender-adjusted plasma betaine concentrations (>60.5 µmol/L) and a “low plasma betaine group” was defined as the bottom quintile of adjusted plasma betaine (<34.1 µmol/L). Dichotomous variables were created, “high plasma betaine” (top quintile 1, all not top quintile 0) and “low plasma betaine (bottom quintile 1, all not bottom quintile 0). These variables were included in Cox proportional hazards regression models, and potential confounders (see “Results” section) added to the models to test their robustness as risk factors.

Similarly, a “High plasma TMAO” group was defined as subjects in the top quintile (>12 µmol/L) of plasma TMAO concentrations, and a dichotomous variable included in models, high TMAO (1) or not high TMAO (0).

**Table 2** summarizes the definitions of all the “High” and “Low” groups used in the analysis.

**Table 2:** High and low plasma concentration cut-off values.

	Upper quintile	Lower quintile
Plasma homocysteine (µmol/L)	17.1	10.0
Plasma betaine (µmol/L)	60.5	34.1
Plasma dimethylglycine (µmol/L)	5.80	2.41
Plasma TMAO (µmol/L)	12.0	2.8

Cut-off values were calculated on the total cohort, “High” concentrations defined as above the top quintile, and “low” concentrations as below the bottom quintile. Betaine and dimethylglycine concentrations are adjusted for a gender difference, data from female subjects increased by a percentage to match male subjects.

Correlations were assessed in the first instance as Pearson’s correlation coefficients. Because many of the variables in this study did not have normal distributions, correlations were also assessed as Spearman’s correlation coefficients, almost invariably leading to the same conclusions. The results reported are Pearson correlation coefficients unless otherwise stated. In regression models, interactions were investigated by including interaction terms (products of the variables).

## Results

### Plasma trimethylamine *N*-oxide (TMAO) stability and distribution

TMAO was measured on plasma specimens collected from 74 subjects with diabetes and 381 subjects without diabetes. Since these had been stored 5 to 8 years before analysis, some at −20°C and some at −85°C, a comparison was made between samples collected from 16 subjects for whom two aliquots had been stored separately, one aliquot at −20°C and the other at −85°C. The TMAO content of these duplicated samples varied from1.6 µmol/L to 29.6 µmol/L. The means and standard deviations in those stored at −20°C was 10.4±8.9 µmol/L; in those stored at −85°C they were 9.9±9.2 µmol/L. The two sets of data were not significantly different (Wilcoxon Signed Rank Test, p = 0.13) and agreed well (r^2^ = 0.95 with the slope of the regression line not significantly different from 1.0 and the intercept not significantly different from 0). We concluded that any small differences attributable to different storage conditions would be negligible compared with the variations between subjects, and data were therefore combined using specimens stored under different conditions.

The median plasma TMAO concentration in subjects with diabetes was significantly (p<0.001, Mann-Whitney Rank Sum Test) higher than in the subjects without diabetes (Table 1).

### Effect of diabetes on prediction of events

**Figure 1:**
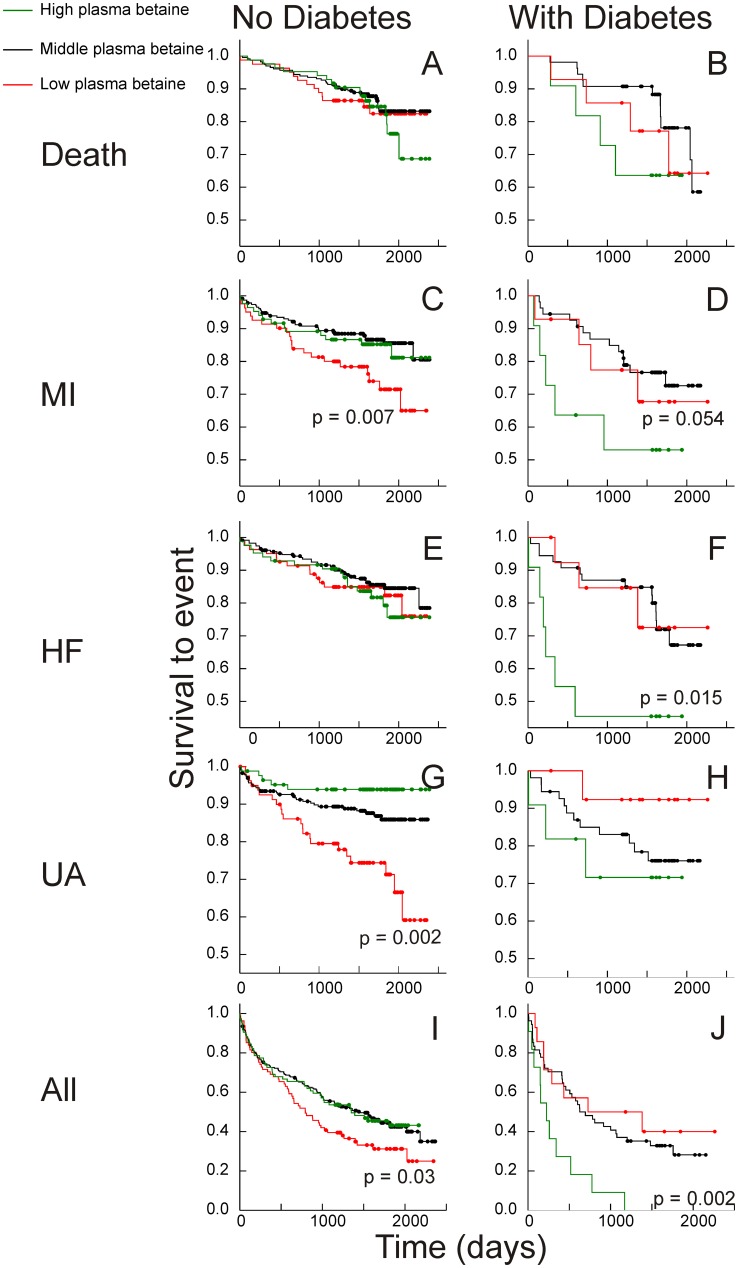
Kaplan-Meier plots: betaine. Plasma betaine concentrations and Kaplan-Meier plots of survival to events. Events: Top (A&B) death from all causes; (C&D) secondary myocardial infarction (MI); (E&F) hospitalization for heart failure (HF); (G&H) unstable angina (UA), and (H&I) all cardiovascular events. On left, A,C,E,G&H are subjects without diabetes; on right, B,D,F,H&J are subjects with diabetes. “High” (green) is the highest quintile of plasma betaine concentration, “Low” (red) the bottom quintile of plasma betaine concentration; “Middle” (black) the remaining 60% of the cohort. All significances are for comparisons with the middle group. Where no significance is shown, p for the difference is >0.3.

When the mean times to secondary events were compared between high (highest quintile) or low (lowest quintile) subjects and the central 60% of the total sample population, the most striking difference was in the predictive significance of plasma betaine (**Fig. 1**). In patients with diabetes, high (top quintile) plasma betaine concentrations were associated with an increased frequency of events when compared with patients with the middle 60% of betaine concentrations; the differences were large for some events, and despite the small number of patients the differences were significant for admissions for heart failure and for all cardiovascular events, with a trend (p = 0.054) in the case of secondary MI. In contrast (Fig. 1), in subjects without diabetes low plasma betaine was more likely to be associated with events, significantly so for MI, unstable angina and for all CVD, and there were no detectable differences in mean survival times between the middle and high plasma betaine groups.

The survival times to events in subjects with elevated plasma DMG were not statistically significantly different between the subjects who have, and those that do not have, diabetes (**Table 3**). The same may be true for plasma homocysteine (Table 3); a possible difference in the predictive value of modest elevations (comparing the bottom quintile with the combined middle three quintiles) for secondary MI needs to be evaluated in a larger study.

**Table 3:** Predictors of events for subjects with and without diabetes.

Survival time (days) to outcome:	Death	MI	HF	UA	All CVD
*With diabetes n = 79*					
Plasma DMG middle	1897	1883	1784	1893	846
Plasma DMG high	1579	**1301**	1344	1415	779
* cf* middle:	(0.15)	**(0.018)**	(0.14)	(0.060)	(0.82)
Plasma DMG low	1865	1799	1912	2033	1256
* cf* middle:	(0.44)	(0.39)	(0.85)	(0.70)	(0.18)
Plasma Hcy middle	2040	1816	1842	1946	1026
Plasma Hcy high	**1351**	**1133**	1280	1587	**391**
* cf* middle:	**(<0.001)**	**(0.013)**	(0.058)	(0.53)	**(0.001)**
Plasma Hcy low	2067	2078	1957	1907	1128
* cf* middle:	(0.52)	(0.058)	(0.41)	(0.86)	(0.46)
*Without diabetes n = 396*					
Plasma DMG middle	2151	2094	2119	2115	1312
Plasma DMG high	2068	1945	**1934**	2066	1131
* cf* middle:	(0.30)	(0.12)	**(0.036)**	(0.94)	(0.14)
Plasma DMG low	2156	2078	**2254**	2128	1333
* cf* middle:	(0.44)	(0.91)	**(0.025)**	(0.83)	(0.75)
Plasma Hcy middle	2199	2124	2171	2098	1329
Plasma Hcy high	**1664**	**1643**	**1422**	**1759**	**874**
* cf* middle:	**(<0.001)**	**(<0.001)**	**(<0.001)**	**(0.019)**	**(<0.001)**
Plasma Hcy low	2135	2210	**2369**	2255	1466
* cf* middle:	(0.099)	(0.44)	**(0.003)**	(0.11)	(0.38)

“High” means top quintile of the total population, “Low” bottom quintile, and “Middle” the central 60% (reference group). Statistics are based on the numbers given in parentheses, which are subjects for which all relevant data is available. MI: secondary myocardial infarction; HF: hospitalization for heart failure; UA secondary unstable angina; all CVD: all cardiovascular events. DMG: *N,N*-dimethylglycine; Hcy: homocysteine.

**Figure 2:**
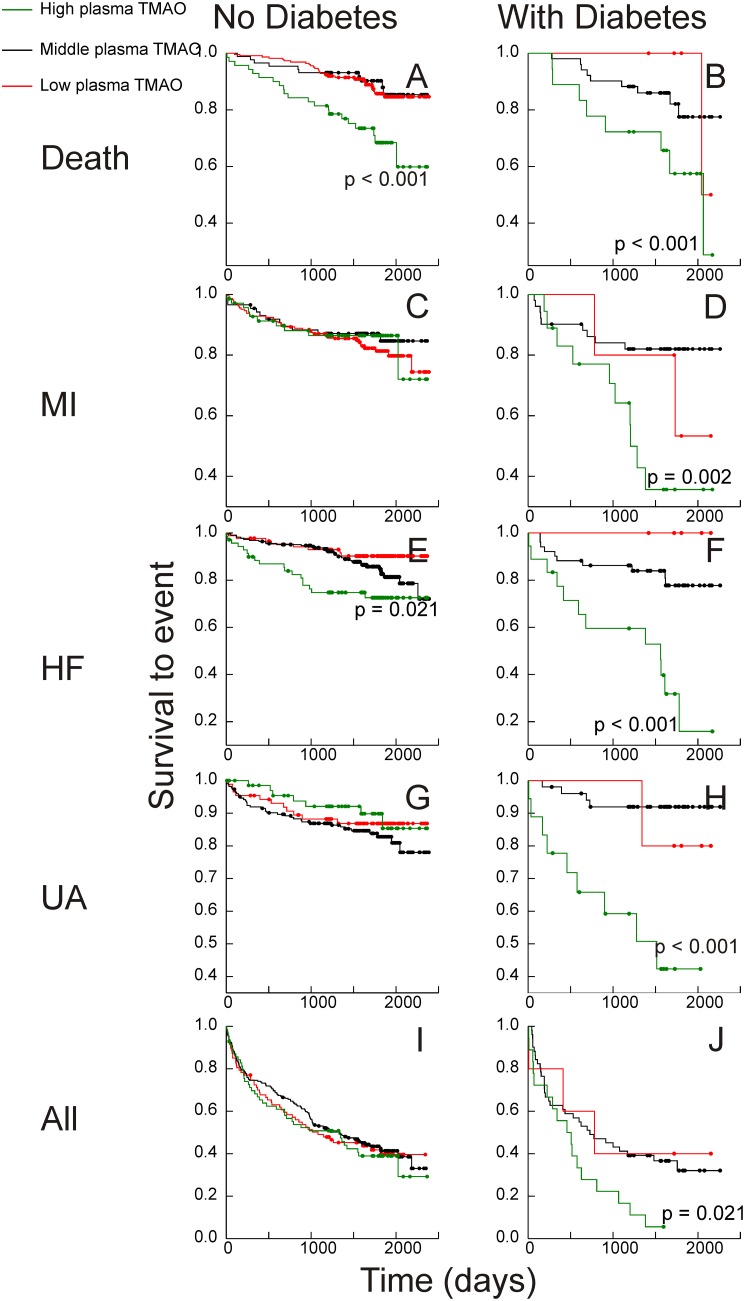
Kaplan-Meier plots: trimethylamine-*N*-oxide. Plasma trimethylamine-*N*-oxide concentrations and Kaplan-Meier plots of survival to events. TMAO: Trimethylamine *N*-oxide. Events: Top (A&B) death from all causes; (C&D) secondary myocardial infarction (MI); middle (E&F) hospitalization for heart failure (HF); (G&H) unstable angina (UA), and (H&I) all cardiovascular events. On left, A,C,E,G&H are subjects without diabetes; on right, B,D,F,H&J are subjects with diabetes. “High” (green) is the highest quintile of plasma betaine concentration, “Low” (red) the bottom quintile of plasma betaine concentration; “Middle” (black) the remaining 60% of the cohort. All significances are for comparisons with the middle group. Where no significance is shown, p for the difference is >0.3.

In both subgroups, elevated (top quintile) plasma TMAO was found to be a significant risk marker for death and hospitalization for heart failure (**Fig. 2**). Sample numbers are shown in Table 1. Because patients with diabetes tend to have elevated plasma TMAO levels (Table 1), the low plasma TMAO data shown in Fig. 2 for patients with diabetes is based on only 5 subjects. While the data was included in Fig. 2 for the sake of completeness, no conclusions can be made about the significance of low plasma TMAO concentrations. Elevated plasma TMAO was a strong risk marker for other cardiovascular outcomes such as MI and unstable angina in the subjects with diabetes (Fig. 2), suggesting that diabetes accentuates the relationship of elevated TMAO and increased cardiovascular risk.

### Hazard ratios

**Figure 3:**
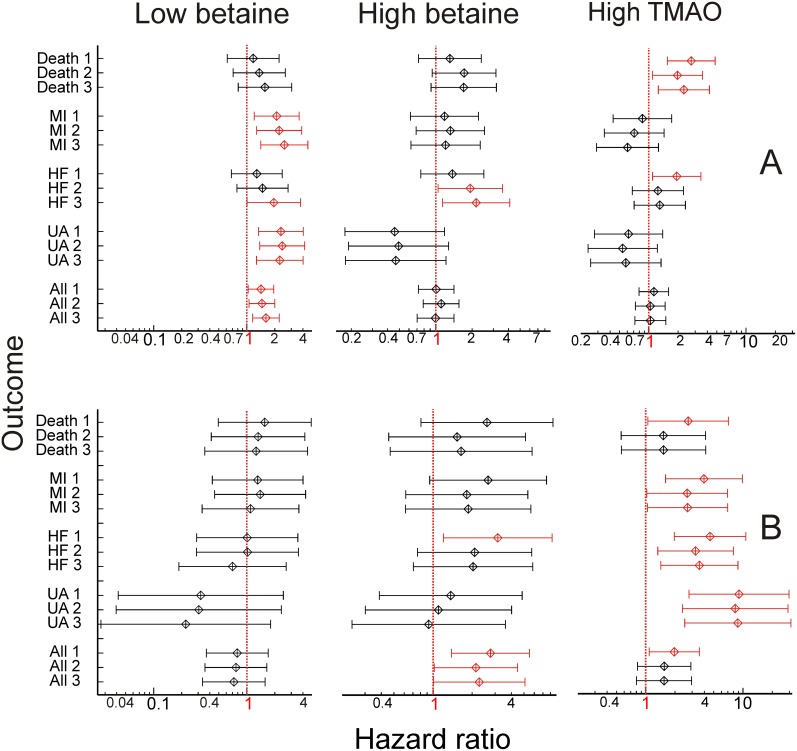
Hazard ratios. Hazard ratios for low (left) or high (middle) plasma betaine concentrations, and for high plasma trimethylamine *N*-oxide concentrations (right). Model 1: just low and high marker (2 variables). Model 2: baseline eGFR added to model. Model 3: Model 2 plus baseline LVEF and plasma NT-proBNP added to model. A: in subjects without diabetes; B: in subjects with diagnosed diabetes. Events: MI: secondary myocardial infarction; HF: hospitalization for heart failure; UA: unstable angina; All: all cardiovascular events. Left side: low plasma betaine; middle: high plasma betaine; right side: high plasma trimethylamine *N*-oxide. Vertical dotted red lines: hazard ratio of 1.0. Ratios significantly (p<0.05) different from 1 are shown in red. All ratios are shown with 95% confidence intervals.

Hazard ratios were estimated to give easy to interpret summaries of the risk associated with high or low (top or bottom quintile) plasma betaine and high (top quintile) plasma TMAO. When “high plasma betaine” (top quintile of gender-adjusted plasma betaine concentrations compared with all other results) and “low plasma betaine” (bottom quintile compared with all other results) were assessed as predictors of secondary outcomes in two-variable Cox proportional hazards regression models, the hazard ratios (**Fig. 3**) were concordant with the Kaplan-Meier analyses (Figs. 1 & 2). High plasma betaine appeared as a significant predictor in the subjects with diabetes, and despite the small numbers the hazard ratios were significant for hospital admission for heart failure (hazard ratio 3.1, CI 1.2–8.2; p = 0.019) and all events (hazard ratio 2.8, CI 1.4–5.5; p = 0.004). In contrast, in the subjects without diabetes, low plasma betaine was a risk marker, notably for secondary myocardial infarction (hazard ratio 2.1, CI 1.2–3.6; p = 0.009), unstable angina (hazard ratio 2.3, CI 1.3–4.0; p = 0.003) and all events (hazard ratio 1.4, CI 1.0–1.9; p = 0.031).

Cox proportional hazards regression models were also calculated for each outcome, with betaine (low and high), relevant covariates and potentially confounding variables included (Fig. 3). Two additional models were tested in each case, one with baseline eGFR (to test whether renal function could explain the apparent hazards) added to the two betaine variables, and one including, along with eGFR, plasma NT-proBNP and LVEF, to assess whether the severity of baseline vascular disease explained the apparent hazards. In the group without diabetes, adding in these potential confounding variables tended to increase the significance of low plasma betaine as a risk marker (Fig. 3). Further models of this group confirmed this; in a model assessing prediction of myocardial infarction that included the plasma betaine dichotomous variables, age, eGFR and NT-proBNP only low plasma betaine (p = 0.001) and NT-proBNP (p<0.001) appeared as significant risk markers, with a hazard ratio for low plasma betaine of 2.5 (1.4–4.4). Potential confounding variables did not attenuate the predictive value of low plasma betaine (Fig. 3), and suggest that for the outcome of hospitalization for heart failure, high plasma betaine may be predictive. Although all data is presented for completeness, there were too few events (Table 1) in the small sample with diabetes to draw negative conclusions from models with more than two variables. In most cases, there were not enough events to justify including more than two or three variables in the models. The reported overall predictive power of the larger models apparently improved, but individual variables became not, or only marginally, significant, and this is true of even well-established risk factors such as plasma NT-proBNP. The lowest p-values were usually associated with the high plasma betaine dichotomous variable. For example, with all CVD events (the largest number of events: Table 1) as the outcome, when eGFR was added to the model (along with high and low betaine), high betaine (p = 0.044) and eGFR (p = 0.045) were still marginally significant risk markers; when NT-proBNP was added as well, these probabilities became 0.063 and 0.108, and even NT-proBNP did not appear to be significant (p = 0.6). It is notable that, despite the small numbers, so many betaine-related factors remain significant (Fig. 3), but a larger study would be needed to confirm this.

Only data for the dichotomous variable “High TMAO” (true when plasma TMAO concentrations were in the top quintile of the total sample, >12.0 µmol/L) is presented, the “Low TMAO” group (n = 5 for subjects with diabetes) was too small to be meaningful (Fig. 3). Cox proportional hazards regression models showed that plasma TMAO was consistently a significant risk marker in the subjects with diabetes, but only for death (all causes) and for hospitalization for heart failure in the subjects without diabetes (Fig. 3). Adding eGFR to the models attenuated the significance of TMAO as a risk marker, but it remained significant in many cases (Fig. 3), despite the sub-optimal number of events. Including high TMAO in models with high or low plasma betaine did not attenuate the apparent significance of high or low plasma betaine concentrations, indeed, the tendency was for these (high betaine in diabetes, low betaine in subjects without diabetes) to be stronger with high TMAO in the models. For example in the with-diabetes sample, high plasma betaine became a significant marker for MI, hazard ratio 3.1 (1.1–8.9), p = 0.04, in a model with high and low plasma betaine and high TMAO.

### Correlations between betaine and metabolites

Although plasma betaine was a significant predictor of plasma DMG in both subgroups (r = +0.52, p<0.001 with diabetes, r = +0.19, p<0.001 without diabetes), the association was significantly (p = 0.002) stronger in the patients with diabetes. Similarly, the association between plasma DMG and homocysteine (r = +0.42, p<0.001 with diabetes, r = +0.18, p<0.001 without diabetes) was stronger (p = 0.034) in the patients with diabetes.

Age was weakly correlated with plasma betaine in the subjects without diabetes (r = +0.13, p = 0.009) but the correlation (r = +0.13, p = 0.23) was not significant in those with diabetes, though in this group with diabetes there was a trend for the dichotomous high betaine variable to be positive with age (r = +0.26, p = 0.021). Conversely, plasma DMG and age correlated in the subjects with diabetes (r = +0.34, p = 0.002) but the correlation was not significant in those without diabetes (r = +0.08, p = 0.13).

The dichotomous high plasma betaine variable correlated with eGFR in both subjects with and without diabetes, but the correlation was positive (r = +0.104, p = 0.038) in the group without diabetes, and negative (r = −0.25, p = 0.028) in those with diabetes; this difference is significant (p = 0.004). Low plasma betaine did not appear to correlate with eGFR in either group. Both high (r = +0.24, p = 0.037) and low (r = +0.27, p = 0.018) plasma betaine positively correlated with homocysteine in the subjects with diabetes, but only low plasma betaine (r = +0.11, p = 0.027) significantly correlated with homocysteine in subjects without diabetes. Correlations between the dichotomous plasma betaine variables and baseline NT-proBNP and with baseline LVEF were not significant except for a probable association between high plasma betaine and NT-proBNP (r = +0.23, p = 0.041) in subjects with diabetes.

No correlations (Spearman’s) between plasma TMAO and plasma betaine were detected, either in the subjects with diabetes or in those without (|r_s_| <0.05). Plasma TMAO may weakly correlate with plasma DMG (r_s_ = +0.17, p = 0.001 without diabetes, +0.18, p = 0.12 with diabetes) but it correlates more strongly with eGFR (r_s_ = −0.31 without diabetes, −0.39 with diabetes; both p<0.001). There were also significant correlations with age (r_s_ = +0.27 without diabetes, +0.38 with diabetes; both p<0.001) and with plasma NT-proBNP (r_s_ = +0.25 without diabetes, p<0.001; +0.35 with diabetes, p = 0.0026). No correlations were detected between plasma TMAO and either baseline ejection fractions or BMI.

### Interaction between betaine and TMAO

The interaction terms between plasma betaine, high-plasma-betaine or low-plasma betaine, and plasma TMAO or high-TMAO were not significant when included in Cox regression models for cardiovascular outcomes. However, the interaction terms between high-plasma-betaine or plasma betaine concentration and TMAO or high-TMAO were significant when included in multiple linear regression models for eGFR in the subjects with diabetes, both with the continuous variables and with the dichotomous variables high-plasma betaine and high-TMAO (p = 0.028). There was no detectable interaction between low plasma betaine and TMAO for predicting eGFR, though it was noted that both high-TMAO (p<0.001 with a negative coefficient) and high-plasma-betaine (p = 0.016 with a positive coefficient) were significant factors in these models.

## Discussion

We have previously observed that plasma betaine concentrations are more variable in patients with diabetes than in the general population [4], suggesting that diabetes affects betaine metabolism. We [11], [32] and others [10] have shown that low plasma betaine concentrations are associated with unfavourable cardiovascular risk markers, and poor outcomes, especially in patients with the metabolic syndrome. Here, we show that in diabetes high plasma betaine concentrations can also indicate an increased cardiovascular risk. High plasma betaine concentrations were reported to mark an increased cardiovascular risk by Wang et al [23], [24], in a population undergoing non-urgent coronary angiography that is not reported to exclude subjects with diabetes.

An observational study of the type presented here cannot, by its nature, define mechanisms: however, these results can suggest further studies to elucidate mechanisms. These considerations must start from understanding the roles of betaine. Betaine is an essential osmolyte that is accumulated in most tissues to regulate cell volume [33], and it also supplies methyl groups to convert homocysteine to methionine [2], a process that generates DMG. Because it has these two roles we can expect that disruption of the supply of betaine to cells will have complex effects. Betaine is obtained directly from the diet, or by mitochondrial oxidation of choline (predominantly in the liver) [1]–[3]. Plasma betaine concentrations are homeostatically controlled [18]–[20] and normally only small amounts of betaine are shed in the urine (fractional clearance <2.5%), even after administration of a betaine load [34], [35]; plasma and urine betaine are not correlated and it follows that betaine homeostasis is regulated through betaine metabolism. The stronger correlations between DMG and both betaine and homocysteine in diabetes could possibly indicate increased metabolism of betaine in diabetes, but because plasma concentrations do not necessarily reflect tissue concentrations [33] caution is needed when making metabolic interpretations of changes in plasma concentrations.

This does not explain why high plasma betaine concentrations in diabetes are a cardiovascular risk marker. Mouse model studies [8] suggest that there may be increased betaine metabolism in diabetes, and this could be expected to lower plasma betaine. Another possible mechanism that could link elevated betaine with pathology might be a decrease in the efficiency of betaine retention by cells in some severely compromised patients. Tissue betaine concentrations are normally much higher than plasma betaine concentrations [33] and efflux is tightly controlled [36], being both osmoregulated and subject to hormonal control (eg by vasopressin and glucagon). It is possible that some subjects with diabetes have elevated plasma betaine concentrations because of a defective control of efflux, for example, as a result of disturbed hormonal control, or as a result of non-enzymic glycation of the poorly characterized membrane proteins that mediate efflux. Since an elevated plasma homocysteine concentration is a marker of betaine deficiency, this interpretation could be consistent with the positive correlation of both high and low plasma betaine with plasma homocysteine: cells leaking betaine may become betaine deficient. There is a strong linkage between betaine metabolism and osmoregulation [37] and disturbances could be expected to have far-reaching consequences. The observation that, when selected confounders are included, high plasma betaine may be a significant risk factor for other cardiovascular endpoints even in the absence of diabetes emphasises the need for further investigations.

Others have reported results that are consistent with those reported here. Svingen et al. [22] found that elevated plasma DMG was an independent risk marker for myocardial infarction in patients with stable angina pectoris in a larger population than that reported here: the differences between that study and the present one include the entry criterion (angina, whereas in this study patients were recruited after hospitalization for a cardiovascular event), which results in the present study subjects being older; and the baseline sample, in the present study baseline samples were collected at an outpatient visit typically about four months after the event rather than at the initial event. Wang et al. [23], [24] reported that elevated plasma betaine was a risk factor for cardiovascular disease, and while these reports are not entirely consistent with other studies, the observations made here may help resolve some of this contradiction. We have identified diabetes as a condition that results in elevated plasma betaine becoming a cardiovascular risk factor, but have not excluded the possibility that other conditions or drugs may have a similar effect, and there are indications (especially in the risk of hospitalization for heart failure) that this is the case.

There has been recent interest in the plasma concentrations of a related substituted trimethylamine, TMAO, as markers of cardiovascular risk [23], [24]. Plasma TMAO has previously been shown to be a marker of renal disease [25], [26], and is a biomarker of fish consumption [38]. In human plasma, the only known source is by the oxidation of trimethylamine [39]; trimethylamine, in turn, is produced by the microbiota in the gut from dietary components. These are not only the TMAO that is found in many fish [38], but also other substituted trimethylamines such as choline [23], [24] and carnitine [40]. TMAO may be cytotoxic in mammals [23], [24], so elevated TMAO may be a cause of disease. In the present study, the association of TMAO with renal disease (marked by eGFR) was confirmed, and this could explain some (but not all) of the risk associated with elevated plasma TMAO. These results do not indicate causes, and the converse possibility that TMAO cytotoxicity is causal in renal disease cannot be excluded. There was a strong interaction between diabetes and plasma TMAO, and high TMAO was only a consistently strong marker of risk for cardiovascular disease in the subjects with diabetes: however, its predictive value for death (all causes) in the subjects without diabetes shows that it cannot be regarded as benign in the absence of diabetes. The observation that plasma TMAO and betaine significantly interact suggests that another possibility that should be investigated is whether TMAO (which is a betaine analog) affects betaine metabolism, for example, by modifying its transport into or out of cells.

The results reported here are mostly consistent with those reported in other studies [22], [24]; the minor differences may reflect differences in the populations studied. Two particular differences are probably important. Firstly, our population is older and in poorer health than in other studies. This results from our recruitment of subjects who have been hospitalized for a coronary disease event. Secondly, our baseline samples were collected at an outpatient clinic after a stabilization period (typically about four months) following the event; this is important since many plasma components (notably plasma betaine [41]) are strongly affected by trauma and samples taken near the time of the event would be expected to give different results. The main weakness of our study is its small size, and in particular there were fewer than 80 subjects with diabetes. Despite this, we show that some of the effects of diabetes on the relationship between betaine-related measures and cardiovascular risk are large enough to be significant, with indications of further trends that we would expect to be confirmed as significant by a larger study. Our study only records secondary events in a population with serious disease: investigations of primary events, and investigations in younger populations with less advanced disease, are also needed. Further, we did not have haemoglobin A_1c_ data available; in future studies it would be desirable to be able to look for relationships with this measure of glycemic control in diabetes. We hope that the results we present here will encourage others to undertake such studies, and to investigate possible causes.

## Conclusions

In diabetes, elevated plasma betaine and trimethylamine-*N*-oxide are risk markers for secondary cardiovascular events. In patients without diabetes low plasma betaine is more likely to be a predictor of secondary events. Betaine metabolism appears to be disturbed in diabetes, and the risks associated with elevated betaine or TMAO in diabetes appear to be associated with renal function.
